# A case report of acute Sheehan syndrome with a review of 29 existing reports from 1990 to 2024: is it still considered a rare disease?

**DOI:** 10.3389/fsurg.2025.1561610

**Published:** 2025-09-29

**Authors:** Chulhyo Jeon, Kiyoung Sung, Jinbeom Cho

**Affiliations:** 1 Department of Surgery, Uijeongbu St. Mary’s Hospital, College of Medicine, The Catholic University of Korea, Seoul, Republic of Korea; 2 Department of Surgery, Bucheon St. Mary’s Hospital, College of Medicine, The Catholic University of Korea, Seoul, Republic of Korea

**Keywords:** hemorrhage, shock, postpartum period, pituitary gland, hormones

## Abstract

**Background:**

Sheehan's syndrome (SS) can occur as a consequence of massive postpartum hemorrhage (PPH). Although traditionally considered rare, acute SS may be underestimated, especially in resource-limited settings, where even minor obstetric complications other than the commonly recognized uterine atony could lead to significant clinical consequences.

**Case report:**

A 32-year-old pregnant woman (gravida 1, para 0) at 37 weeks and 6 days of gestation developed massive PPH complicated by hemorrhagic shock, cardiac arrest, and an emergent hysterectomy, with an estimated blood loss of 20 L. Despite initial stabilization, the patient experienced acute delirium and hyperpyrexia on postpartum day 9. Urgent brain magnetic resonance imaging (MRI) and hormonal studies revealed acute necrosis of the anterior pituitary gland accompanied by multiple endocrine abnormalities. Empirical dexamethasone therapy, initially administered due to clinical suspicion of bacterial meningitis before hormonal study results became available, fortunately resulted in rapid clinical improvement. Although the exact mechanism remains unclear, steroid therapy was successfully tapered and discontinued during hospitalization, allowing the patient to be discharged without ongoing hormonal replacement therapy. Follow-up care revealed continued recovery, with partial empty sella observed on MRI 31months postpartum as a sequela of SS.

**Conclusion:**

Acute SS may be underrecognized clinically, suggesting a higher incidence than previously reported. Immediate hormonal replacement might be crucial when acute SS is clinically suspected, given the delays in confirmatory testing. Careful monitoring of postpartum patients exhibiting an unusual clinical course should be implemented, as it may facilitate the early detection of potentially life-threatening complications such as adrenal crisis or acute endocrine dysfunction.

## 1 Introduction

During pregnancy, the pituitary gland undergoes remarkable enlargement, increasing by up to 136% of its normal size, primarily due to hormonal stimulation associated with elevated estrogen levels (1). Despite this adaptive hypertrophy, the accompanying vascular supply does not always proportionally increase, rendering the gland highly susceptible to ischemic injury (2), and this vulnerability can culminate in Sheehan's syndrome (SS), a devastating form of hypopituitarism caused by ischemic necrosis of the pituitary gland following massive postpartum hemorrhage (PPH) and hypovolemic shock, with contributing factors including arterial vasospasm, disseminated intravascular coagulation, small sella turcica volume, and thrombophilia (3). First identified by Harold Sheehan in 1937 through autopsy findings in women who succumbed to severe PPH (4), SS remains a critical but frequently overlooked complication of childbirth. We recently managed a patient with severe hemorrhagic shock secondary to PPH, a case that presented significant challenges in the timely recognition of acute SS, largely due to the nonspecific and equivocal nature of symptoms indicative of hormonal abnormalities. While it is conceptually possible for SS to present at any time following delivery (5), acute presentations have traditionally been considered rare. Given the potential underestimation of its incidence and its life-threatening nature, we aim to further clarify the clinical characteristics and implications of acute SS through this report and a comprehensive review of the literature.

## 2 Case presentation

**Figure 1:**
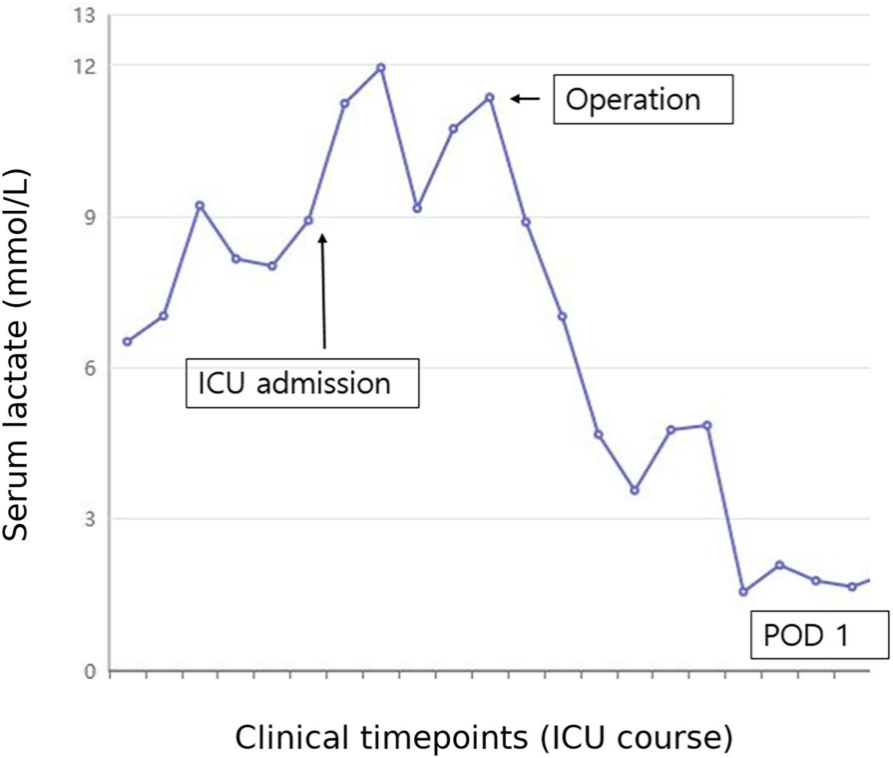
Changes in serum lactate levels during the patient's clinical course in the ICU. The *x*-axis represents sequential blood samples drawn at clinically relevant time points, which are not evenly spaced time intervals. The *y*-axis indicates serum lactate concentration (mmol/L). Serum lactate levels were initially elevated, reflecting ongoing impaired perfusion and hemodynamic instability. Following emergent reoperation (indicated by the arrow), lactate levels markedly decreased, indicating improved perfusion and subsequent clinical stabilization. ICU, intensive care unit; POD, postoperative day.

**Figure 2:**
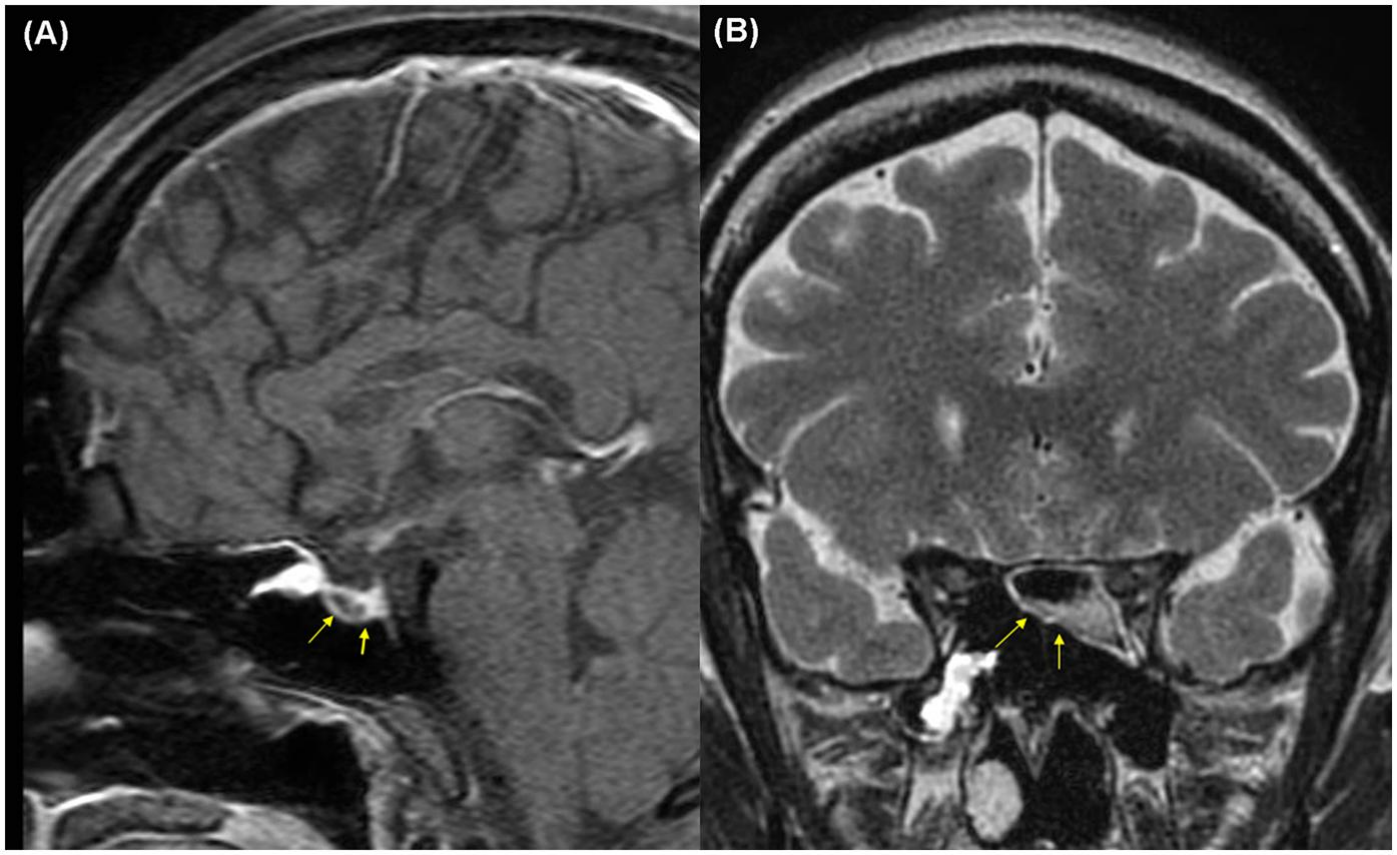
Brain magnetic resonance imaging on postpartum day 9 demonstrating hemorrhagic necrosis of the pituitary gland (yellow arrows): **(A)** sagittal T1-weighted image, **(B)** coronal T2-weighted image.

This report was approved by the Institutional Review Board (IRB) of our institution (HC25ZISI0005), and written informed consent was obtained from the patient. A 32-year-old woman at 37 weeks and 6 days of gestation underwent an elective cesarean section for breech presentation, complicated by massive PPH resulting from uterine atony. The hemorrhage rapidly progressed to hemorrhagic shock and culminated in cardiac arrest. After 20 min of resuscitation, spontaneous circulation was restored, and an emergent hysterectomy was performed. The patient was transfused with 68 units of red blood cells, 80 units of fresh frozen plasma, and 77 units of platelets, along with the infusion of 6,000 ml of crystalloid intraoperatively. Estimated blood loss was approximately 20 L. Upon admission to the intensive care unit, the patient remained in profound shock with ongoing hemorrhage, prompting immediate referral to the acute care surgery department for definitive management of her hemodynamic instability. The patient demonstrated refractory hypoperfusion despite comprehensive organ support, including mechanical ventilation, renal replacement therapy (RRT), and the administration of vasopressors and inotropes at maximal doses. Ongoing hemorrhage was evident through the surgical drain, accompanied by progressive abdominal distension. Consequently, an emergent laparotomy was performed the day after delivery, revealing persistent bleeding from the right adnexa, which was controlled with suture ligation. After surgery, the patient demonstrated a marked improvement in hemodynamic and perfusion parameters (Figure 1), facilitating the cessation of vasopressors on postoperative day (POD) 2, RRT on POD 3, and successful liberation from mechanical ventilation by POD 4. On POD 6, the patient was transferred to the general ward, continued oral feeding, and maintained clinical stability. However, on POD 8 (postpartum day 9), the patient acutely developed delirium, hyperpyrexia peaking at 41°C, tachypnea, and tachycardia. A comprehensive fever workup revealed normal inflammatory markers, including C-reactive protein and procalcitonin, no radiographic evidence of pneumonia, and no significant abnormalities in laboratory investigations, including electrolytes, resulting in considerable diagnostic uncertainty. Nevertheless, based on clinical judgment, a psychotic disorder related to critical illness was empirically suspected, and treatment with antipyretics and haloperidol was initiated; however, these interventions ultimately failed to yield improvement. On that day, despite unremarkable findings on neurological examination and a lower likelihood of bacterial meningitis, the absence of viable treatment options and the critical need to exclude potential life-threatening conditions necessitated prompt initiation of magnetic resonance imaging (MRI) of the brain and cerebrospinal fluid analysis. Simultaneously, empirical treatment with broad-spectrum antibiotics and an intravenous infusion of dexamethasone 10 mg was commenced. Following initiation of treatment, the patient demonstrated rapid and marked clinical improvement, achieving normothermia, restored alertness, and hemodynamic stability by POD 10. Brain MRI demonstrated hypointensity of the anterior pituitary lobe on both sagittal T1-weighted (Figure 2A) and coronal T2-weighted images (Figure 2B). Following gadolinium administration, the gland exhibited peripheral rim enhancement with central non-enhancement, a radiological pattern consistent with acute hemorrhagic necrosis of the adenohypophysis. In light of the clinical course and imaging findings, SS was strongly suspected, and the patient's significant recovery was thought to be attributable to the effects of steroid therapy rather than broad-spectrum antibiotics. Accordingly, hormonal studies were performed to confirm the underlying diagnosis, revealing significant abnormalities in the hypothalamic-pituitary-growth, gonadal, and lactotrophic axes, while thyroid function remained intact, as demonstrated by triiodothyronine, free thyroxine, and thyroid-stimulating hormone levels of 87.52 ng/dl (reference: 80–200 ng/dl), 13.11 pg/ml (reference: 8–20 pg/ml), and 0.90 mIU/L (reference: 0.4–4.0 mIU/L), respectively. The growth axis dysfunction was reflected by a markedly reduced insulin-like growth factor-I level of 78.63 ng/ml (reference: 115–358 ng/ml), while gonadal axis abnormalities were characterized by profoundly low levels of follicle-stimulating hormone at 0.19 mIU/ml (reference range in reproductive age women: 3.5–12.5 mIU/ml, variable by menstrual cycle phase) and estradiol at 1.0 pg/ml (reference range in reproductive age women: 30–400 pg/ml, variable by menstrual cycle phase), with luteinizing hormone at 1.02 mIU/ml, which is inappropriately low for the hypoestrogenic state (reference range in the follicular phase: 2.4–12.6 mIU/ml), consistent with central hypogonadism. Moreover, the lactotrophic axis displayed an inappropriately low prolactin level of 5.17 ng/ml (reference range in pregnancy: typically >100–200 ng/ml). Although the serum cortisol concentration was measured at 20.33 μg/dl (reference range at 8 AM: 5–25 μg/dl), this value should be interpreted with caution. It may have been transiently influenced by the initial administration of hydrocortisone; however, it is also important to note that pregnancy, particularly in the third trimester, is characterized by markedly elevated circulating estrogen levels that increase corticosteroid-binding globulin concentrations, leading to a two- to threefold rise in total cortisol compared with nonpregnant individuals. Therefore, the measured cortisol level in this patient is relatively low for the expected gestational state. In addition, the adrenocorticotropic hormone (ACTH) level (20.6 pg/ml, reference: 10–60 pg/ml), while technically within the normal range, was considered inappropriately low in the context of acute physiologic stress. Collectively, these findings indicate a multifaceted endocrine dysfunction with preservation of thyroid function. During the hospitalization, the patient underwent a gradual tapering of steroid therapy, which was successfully discontinued prior to discharge on POD 17. She was unable to breastfeed after delivery, exhibiting postpartum agalactia, a hallmark clinical manifestation of SS. Subsequently, the patient has been under regular follow-up care with the endocrinology department and remains off any hormonal replacement therapy (HRT) at present. At approximately 31 months postpartum, reassessment demonstrated thyroid function, ACTH, and cortisol levels within normal ranges. Menstrual cycles had not resumed at that time, and hormonal evaluation indicated that the gonadotropic axis remained unrecovered.

## 3 Discussion

Matsuzaki S. et al. published a case report in 2017, summarizing previously reported cases of acute SS (6). At that time, the authors highlighted the rarity of acute manifestations of SS, identifying 19 relevant publications from 1990 to 2014 that included 20 cases, along with their own case, culminating in a total of 21 cases. While their work represented a significant advancement in elucidating the pathophysiology of acute SS, certain aspects were identified as requiring further refinement. In light of this, we sought to build upon their efforts by identifying additional published cases and conducting a more comprehensive review from a clinical perspective. The search was conducted using PubMed, Embase, and Web of Science databases, targeting titles featuring the term “Sheehan” in combination with either “acute” or “early” and limited to publications in English. Studies available only in abstract form were excluded. This process identified 32 cases reported across 29 publications (6–34), and with the inclusion of the present case, a total of 33 cases were documented spanning the period from January 1990 to December 2024. Considering the general tendency not to report cases involving non-survivors, the incidence of acute SS is likely higher than commonly assumed. Detailed information is provided in Supplementary Table S1, with a summary presented in Table 1. Among 33 reported cases, including our own, 20 demonstrated all four key findings of SS: (1) postpartum shock; (2) PPH; (3) endocrine evidence of pituitary dysfunction; and (4) characteristic findings on pituitary imaging. Interestingly, two cases (11 and 12) described peripartum bleeding as routine without clear evidence of significant postpartum hemorrhage. Although these cases do not strictly fulfill the original criteria for SS, they were included because the reporting authors classified them as acute SS based on clinical presentations and hormonal evidence consistent with postpartum hypopituitarism. The remaining cases, despite lacking explicit documentation of one or more diagnostic components, were similarly diagnosed as acute SS by the original authors, aligning with our assessment. The symptoms indicative of SS emerged between 3 h and 28 days postpartum, with a mean onset of 7.64 days and a standard deviation of 6.2 days, while 18 cases (54.5%) occurred within the first 7 days. Neurologic symptoms, including headache, psychosis, seizures, and altered mental status, were the most common initial manifestations, occurring with or without other systemic manifestations, and were reported in 19 cases (57.5%). Additional manifestations included thirst or polyuria in 5 cases, fatigue or asthenia in 5 cases, failure to lactate in 3 case, and respiratory distress in 1 case. Although less critical symptoms may have been overlooked during shock resuscitation, all reported cases showed unusual symptoms emerging only after shock recovery. The most common diagnostic clues suggestive of hormonal abnormalities were hyponatremia (19 cases), followed by hypernatremia (4 cases), hypoglycemia (1 case), urine hypo-osmolality (1 case), and abnormalities in 24-hour urinalysis (1 case). However, in 7 cases, routine laboratory findings failed to provide any clues regarding hormonal abnormalities that could explain the patient's unusual symptoms; in 4 of these cases (13, 19, 20, 23), hormonal studies provided the final diagnosis by revealing evidence of pituitary dysfunction, while brain MRI confirmed the diagnosis in the remaining 2 cases (11, 28) and in our case. Notably, two early reports (7, 8) ascribed hyponatremia to the syndrome of inappropriate antidiuresis (SIAD), although these cases did not meet the diagnostic criteria of SIAD, which require the absence of hypovolemia, edema, or ascites, as well as normal adrenal, thyroid, and renal function. This misclassification was presumably due to confusion between hypothalamic-pituitary-adrenal (HPA) axis dysfunction and SIAD. The patterns of hormonal axis dysfunction showed no consistent trends; however, in 15 cases, the earliest detected abnormality involved the HPA axis. While the posterior pituitary gland is generally considered less vulnerable to ischemic injury than the adenohypophysis due to its direct arterial blood supply, neurohypophyseal dysfunction was reported in 8 cases (11, 12, 16, 22, 24, 26, 30, 33), presenting with symptoms of arginine vasopressin deficiency (formerly known as central diabetes insipidus), such as polyuria and hypernatremia. Notably, in patients with SS, ischemic damage may also affect the hypothalamic thirst center, leading to an increased osmotic threshold for the perception of thirst (35). Reports of axes remaining entirely intact after final hormonal evaluation are rare. However, some literature documents preserved thyrotroph function in 3 cases (9, 31, 32) and our case, gonadotroph function in 3 cases (9, 31, 34), and corticotroph and lactotroph function in 1 case each [26 and 8, respectively]. Among the reported cases, including ours, only two patients were able to discontinue HRT during the follow-up period (10). Notably, no cases reported recovery to a normal pituitary gland on follow-up brain MRI. These findings suggest that permanent HRT might be required in the majority of cases. Nevertheless, periodic reassessment of pituitary function during follow-up is essential, as partial recovery of the HPA may occur in rare instances, allowing adjustment of HRT to optimize long-term quality of life.

**Table 1:** Summary of reported cases of acute Sheehan syndrome from 1990 to 2024.

Reference number	Case number	Year of report	First author	Origin	Age	First symptom	Time to symptom	First diagnostic clue	Finally diagnosed hormonal abnormalities^a^	Components that define Sheehan's syndrome			
										Postpartum hemorrhage	Postpartum shock	Endocrine demonstration for pituitary dysfunction	Imaging evidence
7	1	1991	Putterman C.	Israel	27	Slurred speech, headache	7 days	Hypo-natremia	7	Yes	Yes	Yes	Yes
8	2	1994	Boulanger E.	France	30	Asthenia and failure to lactate	10 days	Hypo-natremia	1,3,4,6	Yes	Not specified	Yes	No
9	3	1995	Zuker N.	South Africa	20	Coma	14 h	Hypo-glycemia	1,5	Yes	Yes	Yes	Yes
10	4	1995	Lavalle G.	Canada	30	Seizure	6 h	Hypo-natremia	1,5	Yes	Yes	Yes	Yes
11	5	1998	Dejager S.	France	32	Headache	5 h	Brain MR	1,2,4,5,6	No	Yes	Yes	Yes
12	6	1998	Kan A.K.S.	Australia	32	Thirst	1 days	Hyper-natremia	2,7	No	No	Yes	No
13	7	1999	Kale K.	India	23	Psychosis	15 days	Hormone study	1,3,4	Not specified	Not specified	Yes	No
14	8	2001	Schrager S.	USA	39	Nausea, vomiting, diarrhea, dizziness, fatigue	12 days	Hypo-natremia	1,3,5	Yes	Suspicious	Yes	No
15	9	2001	Lust K.	Australia	32	Headache	1 days	Hypo-natremia	1,3,6	Yes	Yes	Yes	Yes
16	10	2002	Wang H.Y.	Taiwan	32	Persistent polyuria despite worsening azotemia	7 days	Urine hypo-osmolality	1,2,3,4	Yes	Yes	Yes	No
17	11	2002	Bunch T.J.	USA	23	Fatigue	6 days	Hypo-natremia	7	Yes	Yes	Yes	Yes
18	12	2004	Munz W.	Germany	33	Decreased mentality	6 days	Hypo-natremia	1,3,4,6	Yes	Yes	Yes	No
19	13	2005	Wang S.Y.	Taiwan	33	Respiratory distress	19 days	Hormone study	7	Yes	Yes	Yes	Yes
20	14	2008	Kaplun J.	USA	29	Fever, headache, failure to lactate	17 days	Hormone study	7	Yes	Suspicious	Yes	Yes
20	15				21	Fever, headache	2 days	Hypo-natremia	7	Yes	Yes	Yes	Yes
21	16	2009	Anfuso S.	Italy	35	Asthenia	8 days	Hypo-natremia	7	Yes	Suspicious	Yes	Yes
22	17	2011	Kumar S.	USA	36	Polyuria	4 days	Hyper-natremia	1,2,4	Yes	Suspicious	Yes	No
23	18	2012	Shoib S.	India	31	Psychosis	16 days	Hormone study	1,3,4	Yes	Not specified	Yes	No
24	19	2012	Robalo R.	Portugal	45	Polyuria	15 days	24-hour urinalysis	2,7	Yes	Yes	Yes	Yes
25	20	2014	Sasaki S.	Japan	37	Failure to lactate	4 days	Hypo-natremia	7	Yes	Yes	Yes	Yes
26	21	2014	Hale B.	USA	31	Headache	3 h	Hypo-natremia	2,7	Yes	No	Yes	Yes
27	22	2015	Furnica R.M.	Belgium	28	Asthenia, failure to lactate	2 days	Hypo-natremia	3,4,5,6	Yes	Yes	Yes	Yes
6	23	2017	Matsuzaki S.	Japan	27	Seizure	8 days	Hypo-natremia	7	Yes	Yes	Yes	Yes
28	24	2018	Rahim A.	Australia	27	Headache	2 days	Brain MR	Not specified	Yes	Not specified	Not specified	Yes
29	25	2018	Windpessl M.	Austria	31	Headache	1 day	Hypo-natremia	1,3,6	Yes	Suspicious	Yes	Yes
30	26	2021	Olmes G.L.	Germany	28	Polyuria	2 days	Hyper-natremia	1,2,3,4	Yes	Yes	Yes	Yes
31	27	2022	Pineyro M.M.	Uruguay	34	Headache	7 days	Hypo-natremia	1,5,6	Yes	No	Yes	Yes
32	28	2023	Ishikawa S.	Japan	32	Fatigue, headache	9 days	Hypo-natremia	1,5,6	Yes	Yes	Yes	No
32	29				31	Fatigue, vomitting, impaired consciousness	8 days	Hypo-natremia	1,3,6	Yes	Yes	Yes	Yes
32	30				39	Fatigue, general malaise	9 days	Hypo-natremia	1,3,6	Yes	Yes	Yes	Yes
33	31	2024	Occhiali E.	France	37	Psychosis	8 days	Hyper-natremia	Not specified	Yes	Yes	Suspicious	Yes
34	32	2024	Saito W.	Japan	37	Anorexia	28 days	Hypo-natremia	1,3,5,6	Yes	Yes	Yes	Yes
Present study	33		Chulhyo J.	Korea, Republic of	32	Fever, delirium	9 days	Brain MR	4,5,6	Yes	Yes	Yes	Yes

a Each number corresponds to a specific dysfunction as listed below: (1) Corticotroph; (2) Posterior pituitary; (3) Thyrotroph; (4) Gonadotroph; (5) Somatotroph; (6) Lactotroph; (7) Panhypopituitarism.

In conclusion, our findings and review of the literature suggest that acute SS may be more common than previously recognized. Clinicians should maintain a high index of suspicion for SS in postpartum patients presenting with nonspecific or unusual clinical symptoms, even when bleeding appears clinically manageable. Diagnosing and managing acute SS remain particularly challenging due to diagnostic delays associated with hormonal evaluations and brain MRI. As emphasized by Taher et al. (36), deep learning techniques offer significant potential to enhance clinical decision-making in scenarios characterized by limited or heterogeneous data. Such technological approaches could also aid in the early prediction and recognition of rare yet serious obstetric complications, including acute SS. Additionally, a recent case report (37) underscores the importance of early recognition and prompt initiation of hormone replacement therapy to improve patient outcomes. Empirical initiation of hormone replacement therapy prior to confirmatory diagnostic results may be beneficial in preventing adverse clinical outcomes. Finally, we believe that a significant number of postpartum patients may remain unreported and experience fatal outcomes due to delayed diagnosis. Among the 33 reported cases, 8 involved patients who were discharged postpartum but were later readmitted with symptoms associated with SS (11, 13, 14, 18, 20, 21, 26, 31). Although these cases likely survived due to mild hormonal abnormalities, the risk of life-threatening complications such as adrenal crisis or acute endocrine shock underscores the critical importance of careful monitoring in postpartum patients.

## Data Availability

The original contributions presented in the study are included in the article/Supplementary Material, further inquiries can be directed to the corresponding author.
